# Uptake of extracellular vesicles into immune cells is enhanced by the protein corona

**DOI:** 10.1002/jev2.12399

**Published:** 2023-12-20

**Authors:** Laura Dietz, Jennifer Oberländer, Ana Mateos‐Maroto, Jenny Schunke, Michael Fichter, Eva‐Maria Krämer‐Albers, Katharina Landfester, Volker Mailänder

**Affiliations:** ^1^ Department of Dermatology University Medical Center Mainz Mainz Germany; ^2^ Max Planck Institute for Polymer Research Mainz Germany; ^3^ Institute of Developmental Biology and Neurobiology Johannes Gutenberg University of Mainz Mainz Germany

**Keywords:** protein corona, cell uptake, immune cell, macrophage, exosome, extracellular vesicle

## Abstract

The influence of a protein corona on the uptake of nanoparticles in cells has been demonstrated in various publications over the last years. Extracellular vesicles (EVs), can be seen as natural nanoparticles. However, EVs are produced under different cell culture conditions and little is known about the protein corona forming on EVs and its influence on their uptake by target cells. Here, we use a proteomic approach in order to analyze the protein composition of the EVs themselves and the protein composition of a human blood plasma protein corona around EVs. Moreover, we analyze the influence of the protein corona on EV uptake into human monocytes and compare it with the influence on the uptake of engineered liposomes. We show that the presence of a protein corona increases the uptake of EVs in human monocytes. While for liposomes this seems to be triggered by the presence of immunoglobulins in the protein corona, for EVs blocking the Fc receptors on monocytes did not show an influence of uptake. Therefore, other mechanisms of docking to the cell membrane and uptake are most like involved, demonstrating a clear difference between EVs and liposomes as technically produced nanocarriers.

## 1 INTRODUCTION

Since extracellular vesicles (EVs) are attracting more and more attention as therapeutic agents and drug delivery vehicles, investigating the interaction with blood components becomes increasingly important. Liposomes, which have already entered clinical use as drug delivery vehicles, are the class of synthetic nanoparticles closest to EVs and their protein corona is well investigated. Therefore, we have chosen liposomes for comparison with EVs in this study.

The concept of protein corona formation around synthetic nanoparticles upon blood contact is well established. Numerous studies investigated the composition of proteins forming around synthetic nanoparticles and their influence on biodistribution and cell uptake (Monopoli et al., 2012; Walczyk et al., 2010). For EVs, which can be considered biological nanoparticles similar to liposomes, the protein corona is poorly understood. However, EVs are of interest as therapeutic agents and drug delivery platforms. This raises the need for understanding interactions between EVs and blood components, which ultimately determines cellular uptake and biodistribution (Herrmann et al., 2021; Murphy et al., 2019) also in comparison to liposomes which can be considered as possible synthetic analogues to EVs. In this study, we investigate the influence of a protein corona on HCT 116 cell‐derived tumour EVs on their uptake in phagocytes and the cell line of EV origin in comparison to two types of liposomes.

EVs attracted a lot of interest as drug delivery platforms as they function as communication systems by transporting biologically active cargoes, such as proteins and RNA molecules, even to distant target cells (Herrmann et al., 2021; van Niel et al., 2018). Therefore, the payload is contained in a double‐membrane vesicle that is equipped with transmembrane proteins capable of navigating biological environments including cell targeting and entry. EVs describe a group of vesicles ranging in diameter from 30 to 300 nm that can be subdivided into exosomes, microvesicles, and larger apoptotic bodies. For long‐distance information transport, exosomes and microvesicles are of interest as vehicles. Exosomes are derived from endosomal compartments named multivesicular bodies (MVBs), whereas microvesicles directly bud from plasma membranes (Théry et al., 2018, 2002). During long‐distance transport, EVs encounter biological fluids, such as blood, and proteins likely adsorb to their surface. This is referred to as protein corona formation. The adsorbed protein corona can ultimately alter the EV functionality as observed for many nanoparticles. This has been largely neglected in the field of EV research. The formation of an EV protein corona was first described by Tóth et al. who revealed the protein composition as well as demonstrated the influence of a protein corona on EV functionality (Tóth et al., 2021). Shortly after, this was confirmed by Wolf et al. who demonstrated that a protein corona is necessary for EVs to reach their full potential in angiogenesis and wound healing assays (Wolf et al., 2022). These findings started a paradigm shift from viewing plasma‐derived proteins as contaminants of EV preparations towards considering them as additional EV components.

Presumably, the insights into the protein corona formed on nanoparticles also will benefit the functional understanding of the EV corona. Nanoparticles quickly adsorb proteins upon contact with biological fluids. This changes their chemical identity to a biological identity and ultimately alters their biodistribution and cell uptake (Walczyk et al., 2010). Distinct proteins regulate the uptake of nanoparticles. Apolipoproteins, a class of lipid‐binding proteins abundant in human blood, are known to promote a prolonged blood circulation time by avoiding uptake in phagocytic monocytes of the mononuclear phagocytic system (MPS) (Gustafson et al., 2015). This has also been referred to as stealth effect. In contrast, so‐called opsonins, such as immunoglobulins mark nanoparticles for uptake in phagocytes (Yang et al., 2013). The protein corona on liposomes and lipid nanoparticles is well studied as this is one of the most commonly used drug carrier systems in clinical use (Onishchenko et al., 2021). A major drawback for the clinical application of liposomes still is the formation of an opsonizing protein corona and uptake by phagocytes. Therefore, modifications like attachment of polyethylene glycol (PEG) are used to prolong the liposomes’ blood circulation time (Blanco et al., 2015; Torchilin, 2005).

EVs are a special type of nanoparticle with a biological origin. Hence, they carry integrated and partially integrated proteins by themselves but can also acquire a protein corona from the blood plasma, as just recently demonstrated empirically (Tóth et al., 2021; Wolf et al., 2022). In the previous EV protein corona studies, stem cell and stromal cell‐derived EVs, which are of interest for regenerative and immunomodulatory therapies, were investigated. Here, we investigate the protein corona of tumour‐derived exosomes (TEXs) as drug delivery vehicles analogous to synthetic drug carriers.

TEXs exert various functions in tumour progression and regulation leading to a multitude of possible treatment options using TEX. They are capable of reprogramming the tumour microenvironment by altering the function of neighbouring tumour cells, infiltrating immune cells, and stromal cells (Dai et al., 2020). Additionally, TEXs confer signalling in a long‐distance manner addressing metastases. By this mechanism, chemoresistance and oncogenes are horizontally transferred to other cancer cells and distant metastases (Al‐Nedawi et al., 2008; Asare‐Werehene et al., 2020; Chen et al., 2014). This finding also suggests that TEXs have a certain targeting capacity for tumour cells they originated from. This principle was utilized as a targeting strategy to deliver anti‐cancer drugs (Qiao et al., 2020; Yong et al., 2019). As another treatment option, TEXs play a crucial role in tumour immunity, which is highly dependent on the specific context. There is evidence, that TEX mediates immunosuppression via inhibition of macrophages, dendritic cells (DC), lymphocytes and other immune cells in the tumour microenvironment (Dai et al., 2020; Naseri et al., 2020). On the other side, isolated TEX were successfully used as tumour antigens for DC‐loading and in vivo tumour vaccination (Bu et al., 2006; Gu et al., 2015; Lee et al., 2012; Liu et al., 2017; Wolfers et al., 2001).

Here, we analyzed the protein corona of HCT 116 EVs and its influence on vesicle uptake in comparison to liposomes as model drug carriers. EVs as well as liposomes showed an increased uptake into phagocytic THP1 cells and monocyte‐derived DCs (moDCs) upon protein corona formation. The advantage of proteomics studies of the protein corona is that specific proteins can be determined to explain these effects. Here we demonstrate that enhanced uptake into these cells can be attributed to immunoglobulins and complement protein enrichment in the protein corona. However, uptake via Fc receptors was not the major route for EV uptake upon protein corona adsorption.

EVs and liposomes showed increased uptake also in HCT 116 cells. Our data demonstrates the importance of the protein corona for cell uptake and suggests it represents a critical factor for the application of EVs as drug‐delivery vehicles. Further, we propose that enhanced uptake of EVs by phagocytes in the presence of a protein corona can be a benefit for the application of EVs in immunotherapy.

## 2 MATERIAL AND METHODS

### 2.1 Cell culture

THP‐1 cells were cultured in RPMI 1640 medium supplemented with 10% FBS, 100 U mL^−1^ penicillin, 100 mg mL^−1^ streptomycin, and 2 mM Glutamax. Human colorectal tumour (HCT) 116 cells were cultured in Dulbecco′s Modified Eagle′s Medium (DMEM)—high glucose supplemented with 10% FBS, 100 U mL^−1^ penicillin, and 100 mg mL^−1^ streptomycin. Cells were grown in a humidified incubator at 37°C and 5% CO_2_. For passaging of THP‐1 cells, cells were centrifuged at 300 g for 5 min. For passaging of HCT cells, 0.25% Trypsin‐EDTA was used at 37°C and 5% CO_2_ for 5 min before centrifugation at 300 g for 5 min (all reagents from Thermo Fisher Scientific, Waltham).

### 2.2 Generation of monocyte‐derived dendritic cells

Buffy coats, received from healthy donors upon informed consent (blood bank of the University Medical Center Mainz) were used to isolate peripheral blood mononuclear cells (PBMCs) (Fichter et al., 2015). PBMCs were isolated from other blood cells by centrifugation for 20 min at 900 g at room temperature through a Histopaque‐1077 density gradient media (Sigma‐Aldrich). Subsequently, PBMCs were collected from the interphase and washed with PBS. CD14^+^ monocytes were enriched using magnetic cell separation via CD14 MicroBeads (MACS) (Miltenyi Biotec., Bergisch Gladbach, Germany). The cells were subsequently cultured at a concentration of 10^6^/mL in 3 mL in 6‐well plates (Greiner, Kremsmünster, Austria) using IMDM medium supplemented with 10% FBS, 1% GlutaMAX, 1 β‐mercaptoethanol, 1% non‐essential amino acids, 1 mM sodium pyruvate, 100 U mL^−1^ penicillin and 100 g mL^−1^ streptomycin. Further, 200 U mL^−1^ granulocyte‐macrophage colony‐stimulating factor (GM‐CSF) and 200 U mL^−1^ interleukin‐4 (IL‐4) were added to each well. Before cell uptake, cells were cultured for 6 days at 37°C and 5% CO_2_. 1 mL of culture medium was carefully removed from the wells and replenished with 1 mL fresh medium supplemented with 600 U mL^−1^ GM‐CSF and 600 U mL^−1^ on day 2 and day 4.

### 2.3 EV isolation from the cell culture medium

30 mL of conditioned medium (CM) was collected from approximately 2 × 10^7^ sub‐confluent HCT cells cultured in serum‐free medium for 24 h. CM was centrifuged at 3000 g at 4°C for 20 min. Supernatants were collected and concentrated 20‐fold using Amicon Ultra‐15 30 kDa MWCO centrifugal filters (Merck Millipore, Darmstadt, Germany). EVs were isolated by size exclusion chromatography (SEC) according to Brahmer et al. (2019). 10 mL syringes were packed with Sepharose CL‐2B (Thermo Fisher Scientific). Columns were washed with one column volume PBS before applying 2 mL of concentrated and precleared CM. Fractions of 1 mL were collected and analyzed for particle count by nanoparticle tracking analysis. Fractions 4–6 were collected as particle‐rich fractions and concentrated 30‐fold using Amicon Ultra‐2 30 kDa MWCO centrifugal filters (Merck Millipore, Darmstadt, Germany). Protein content was measured using Pierce 660 nm Protein Assay Reagent (Thermo Fisher Scientific). Absorption was measured at 660 nm with a M1000 plate reader (Tecan, Männedorf, Switzerland).

### 2.4 Liposome preparation

Liposomes were prepared from mixtures of cholesterol (Chol), 1,2‐Dioleoyl‐*sn*‐glycero‐3‐phosphoethanolamine (DOPE), and L‐*α*‐phosphatidylcholine (egg PC) with different molar compositions to reach two different liposome formulations, DOPE 5% and DOPE 33%. The three liposome components were prepared at a concentration of 10 mg mL^‐1^ in chloroform and stored at −20°C. Then, the different liposome membrane compositions were obtained by mixing 1.44 mL eggPC, 120 µL DOPE, and 440 µL Chol, to achieve eggPC:DOPE: Chol = 55:5:40 molar ratio; and 835 µL eggPC, 767 µL DOPE, and 398 µL Chol, to get eggPC:DOPE:Chol = 1:1:1 molar ratio. All chemicals were purchased from Merck, Darmstadt, Germany. The lipid mixtures together with 2 mL of chloroform (Merck, Darmstadt, Germany) with 1 vol% EtOH (Carl Roth, Karlsruhe, Germany) were added in a 50 mL round‐bottomed flask. To obtain the dried lipid films the solvent was evaporated with a rotary evaporator (450 mbar and 3 mbar each for 30 min at 42°C).

The lipid films were hydrated by the addition of 4 mL of PBS buffer (0.1 M, pH = 7.4) (Merck, Darmstadt, Germany). Then the mixtures were stirred overnight at 500 rpm and subsequently sonicated in a water bath for 20 min. Finally, the liposomes were extruded 11 times per pore size through polycarbonate membranes with pore sizes of 800 nm, 400 nm, and 200 nm using a 1 mL syringe (Avanti Polar Lipids, Alabaster). Liposomes were stored at 4°C until further use.

### 2.5 Labelling HCT 116 EVs and liposomes with Cyanine 5 (Cy5)

100 µg of freshly isolated HCT EVs or liposomes were incubated with 4 µL 1 mM Cy5 NHS‐Ester (Lumiprobe, Hunt Valley) at room temperature for 1 h. Unreacted dye molecules were removed by washing the samples with PBS using Amicon Ultra‐2 30 kDa MWCO centrifugal filters (Merck Millipore, Darmstadt, Germany). For labelling of EVs, an excess of fluorophores was used.

The labelling reaction was done in 100 µL at a working concentration of 40 µM Cy5‐NHS. That corresponds to 2.4 × 10^15^ available fluorophore molecules. 100 µL of isolated EVs contained approximately 5 × 10^9^ particles. Therefore, the labelling reactions were performed at a fluorophore excess of 4.8 × 10^5^. Considering that there are multiple available NH2 groups per EV particle, it is likely that at this ratio there was an excess of fluorophores over NH2 groups. To verify that all unbound fluorophores have been washed out to an extend that they do not affect cell uptake studies, a control containing only fluorophores but not particles was prepared. Therefore, the same amount of fluorophore was incubated with PBS and washed according to the staining protocol. Incubation of this sample with HCT 116 cells did not result in an intracellular uptake signal above cell background in flow cytometry measurements.

### 2.6 Dynamic light scattering (DLS)

For DLS measurements, 10 µL of liposome sample were diluted in 200 µL PBS buffer. For EV measurements, 200 µL of undiluted sample was used. Measurements were performed using a Zetasizer Nano S90 (Malvern Panalytical GmbH, Germany) at 25°C. The size distribution was obtained by cumulants fitting.

### 2.7 Nanoparticle tracking analysis (NTA)

For NTA measurments, the ZetaView (Particle Metrix GmbH, Germany) and the corresponding software ZetaView version 8.05.16 SP3 (Particle Metrix GmbH, Germany) were used. The following instrument parameters were used: Scatter mode with a sensitivity of 80 and a shutter of 100. For analysis a max area of 1000, a min area of 10 and a min brightness of 30 were chosen. The binning was set to 5 nm. Samples were diluted with PBS and measurements were done at 25°C. The particles of 11 long videos and a total of 959 objects were analyzed and mean and standard deviation were calculated from number‐weighted mean values.

### 2.8 Zeta potential

A Zetasizer Nano Z (Malvern Panalytical GmbH, Germany) with disposable folded capillary cells was used to determine the zeta potential of the EV and liposome samples. For the measurements, 10 µL of each liposome dispersion or 100 µL of EV solution were diluted with 1 mL of a 1 mM potassium chloride (KCl) solution. The measurement was performed at 25°C after 2 min of equilibration. Each measurement was performed in triplicates and mean values as well as standard deviations were calculated.

### 2.9 Protein corona adsorption

Human citrate blood plasma was taken from healthy donors at the Department of Transfusion Medicine at the University Medical Centre Mainz after physical examination and after obtaining written informed consent in accordance with the Declaration of Helsinki. The blood plasma of 10 healthy donors was pooled and stored at −20°C. The study was approved by the local ethics committee “Landesärztekammer Rheinland‐Pfalz” (Bearbeitungsnummer: 837.439.12 (8540‐F)). Protein corona was adsorbed as previously described by our group (Kokkinopoulou et al., 2017; Simon, Müller et al., 2018; Simon, Wolf et al., 2018). The protocol was adapted to EV properties. 100 µg of Cy5‐labelled or unlabelled EVs or liposomes were incubated with 1 mL of human citrate plasma at 37°C under constant agitation (300 rpm) for 1 h. For EV proteome analysis 100 µg EVs were incubated with 1 mL PBS. Subsequently, EVs and liposomes were pelleted at 100,000 g at 4°C for 1 h with an Avanti J‐26S XP ultracentrifuge (Beckman Coulter, Pasadena). For mass‐spectrometry analysis of the protein corona, pellets were washed 3x with PBS and subjected to digestion for liquid chromatography‐mass spectrometry (LC‐MS). For cell uptake experiments pellets were washed 1x in PBS and the final pellets were resuspended in 1 mL cell culture medium.

### 2.10 Flow cytometry and cytotoxicity

Cell uptake of EVs and liposomes with and without protein corona into THP‐1 cells, HCT 116 cells, and moDCs was measured by flow cytometry. Therefore, 150,000 cells per well were seeded in 24‐well plates (Greiner Bio‐One, Frickenhausen, Germany) and directly incubated with EVs or liposomes (10 µg mL^−1^) at 37°C and 5% CO_2_ for 16 h. Subsequently, cells were centrifuged at 300 g for 5 min and resuspended in 1 mL PBS for flow cytometry measurement. For binding assays in the absence and presence of Fc receptor block, 100,000 cells were pretreated with 0.5 µL Human TruStain FcX (Biolegend, San Diego) for 10 min at room temperature. Subsequently, cells were centrifuged at 300 g for 5 min and resuspended in 10 µL cell culture medium without serum. Diluted EV or liposome solution (20 µg mL^−1^) was added and incubated with cells at 4°C for 40 min. Subsequently, cells were centrifuged at 300 g for 5 min and resuspended in 1 mL PBS for flow cytometry measurement. Nanoparticle uptake was measured with an Attune NxT Flow Cytometer (Thermo Fisher Scientific). Measurements were stopped, after reaching 10,000 events for the cells. Cy5 signal of EVs or liposomes was detected in the RL1 channel with excitation at 638 nm and a 670/14 nm band pass filter. The Attune NxT Software was used for data analysis. FSC/SSC scatter plots were used for cell population selection and events were depicted as percentage of gated cells and median fluorescent intensities. For cell viability measurement, 5,000 cells per well were seeded in 96‐well plates (Greiner Bio‐One, Frickenhausen, Germany) and were directly incubated with EVs or liposomes (10 µg mL^−1^) at 37°C and 5% CO_2_ for 16 h. As dead cell control, cells were incubated with a medium containing 20% dimethyl sulfoxide (Sigma‐Aldrich, St. Louis) for 16 h. CellTiter‐Glo Luminescent Cell Viability Assay (Promega, Madison) reagent was prepared according to manufacturer's protocol and then 100 µL reagent was added per well. Luminescence was measured with an M1000 plate reader (Tecan, Männedorf, Switzerland).

### 2.11 Confocal laser scanning microscopy (cLSM)

The intracellular localization of liposomes and EVs in THP1 cells was verified by cLSM images taken with a Leica TCS SP8 (Leica, Wetzlar, Germany). The microscope was equipped with a multi‐laser combination and five detectors (range of 400–800 nm). Cy5‐labelled liposomes and EVs were excited at 633 nm and a detector range of 646–799 nm. CellMask™ Plasma Membrane Stain Orange (Thermo Fisher Scientific) was excited at 561 nm and detected at 578–618 nm. LysoTracker™ Green DND‐26 (Thermo Fisher Scientific) was excited at 488 nm and detected at 504–541 nm. Images were taken in sequential mode using the LAS X software. For post‐processing, Image J was used, and brightness and contrast settings were adjusted to account for differences in liposome and EV fluorescent labelling. For + and –PC images of the same particle, identical brightness, and contrast settings were used.

150,000 THP1 cells were seeded per well in 48‐well plates (Greiner Bio‐One, Frickenhausen, Germany) and directly incubated with EVs or liposomes (120 µg mL^−1^) at 37°C and 5% CO_2_ for 16 h. Cells were centrifuged at 300 g for 5 min and stained with CellMask and LysoTracker shortly before imaging. For imaging, 10 µL cell suspension was applied to a High Precision Microscope Cover Glass 24 × 60 mm 170 ± 5 µm in thickness (Carl Roth, Karlsruhe, Germany) and covered with a second cover glass 170 ± 5 µm in thickness and 24 mm in diameter (Carl Roth, Karlsruhe, Germany).

### 2.12 Cryo‐transmission electron microscopy (TEM)

CryoTEM samples were prepared on glow‐discharged 300 mesh 2/1 Quantifoil grids (Quantifoil Micro Tools, Großlöbichau, Germany). 4 µL of sample was added to the grid and blotted using Vitrobot Mark (Thermo Fisher Scientific) for 1 sec using force 5 at 20°C in 100% humidity. The grid was then plunge‐frozen in liquid ethane and imaged with a K3 camera (Gatan, Pleasanton) in the Titan G4 Cryo‐TEM (Thermo Fisher Scientific). For statistical analysis of the vesicle diameter distribution, five images and a total of 34 objects were taken into account. Vesicle diameters were measured by hand using Image J.

### 2.13 In‐solution digestion and LC‐MS measurement

The in‐solution digestion and LC‐MS measurements were performed as previously described by our group (Kokkinopoulou et al., 2017; Simon, Müller et al., 2018; Simon, Wolf et al., 2018). In short, SDS was removed by using Pierce Detergent Removal Spin Columns (Thermo Fisher Scientific) followed by protein precipitation using ProteoExtract protein precipitation kit (Merck Millipore, Darmstadt, Germany). After isolation, proteins were resuspended in RapiGest SF (Waters, Milford), reduced with dithiothreitol (Sigma‐Aldrich, St. Louis), and alkylated with iodoacetamide (Sigma‐Aldrich, St. Louis). Tryptic digestion of the protein was performed at a protein:trypsin ratio of 50:1 for 18 h at 37°C. After stopping the digestion by adding 2 µL HCl (Sigma‐Aldrich, St. Louis) and removing the degradation products of RapiGest by centrifugation, the peptides were proceeded to LC‐MS measurements. Therefore, the samples were diluted with 0.1% formic acid and spiked with 50 fmol µL^−1^ Hi3 E. coli (Waters, Milford). Measurements were performed at a nanoACQUITY UPLC system coupled to a Synapt G2‐SI mass spectrometer (Waters, Milford). Electrospray ionization was performed in positive mode with a NanoLockSpray source. As a reference, Glu‐Fibrinopeptide (150 fmol µL^‐1^) at a flow rate of 0.5 µL min^−1^ was injected and a sample flow rate of 0.3 µL min^−1^ was set. Mass spectrometer was operated in a resolution mode performing data‐independent acquisition (MSE). Data was processed using MassLynx 4.1 and proteins were identified using Progenesis QI 2.0. A reviewed human database downloaded from Uniprot was used for protein identification. Noise reduction thresholds were set for low energy, high energy, and peptide intensity at 120, 25, and 750 counts, respectively. A maximum protein mass of 600 kDa, one missed cleavage, fixed carbamidomethyl modification for cysteine, variable oxidation for methionine, and a false discovery rate of 4% for proteins was set for protein and peptide identification. For peptide identification at least three assigned fragments and for the protein identification at least two assigned peptides and five assigned fragments are required. The TOP3/HI3 approach was used for the quantification of each protein in fmol (Silva et al., 2006).

## 3 RESULTS AND DISCUSSION

**FIGURE 1:**
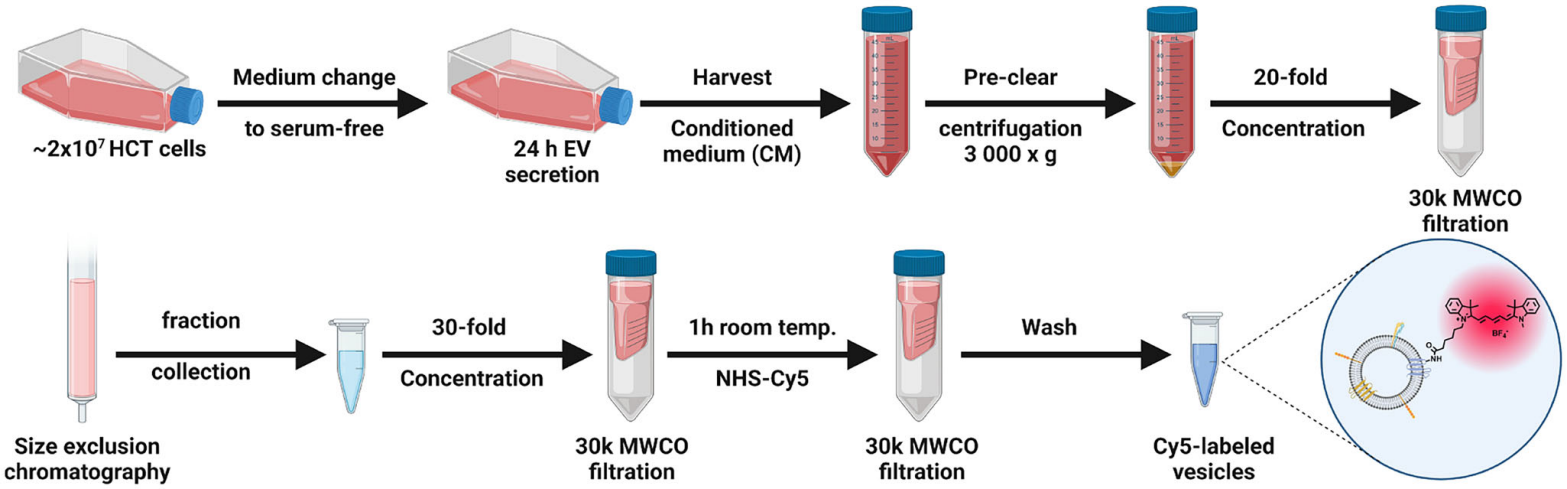
Production of HCT 116 EVs and Cy5‐labelling. EVs were collected under serum‐free conditions for 24 h and subsequently purified via SEC. After concentration, EVs were labelled by reacting NHS‐coupled Cy5 with primary amine groups of EV surface proteins in aqueous solution. Access dye is removed by washing with 30k MWCO centrifugal filters.

Here, we used HCT 116‐derived tumour EVs and compared them to similar‐sized liposomes as synthetic analogue. The EVs were collected under serum‐free conditions to avoid a pre‐formed serum protein corona. EVs were isolated by size exclusion chromatography (SEC) to separate EVs from ECM components and other proteins secreted by the producing cell. This separation method isolates vesicles based on size. Therefore, the resulting sample contained a heterogeneous mixture of exosomes, microvesicles and other vesicles. In addition, SEC is considered to be minimally damaging to the EVs’ surface which preserves EV functionality (Böing et al., 2014; Sidhom et al., 2020). Subsequently, EVs were labelled by reacting NHS‐Cy5 to primary amines of EV surface proteins (Figure 1).

**FIGURE 2:**
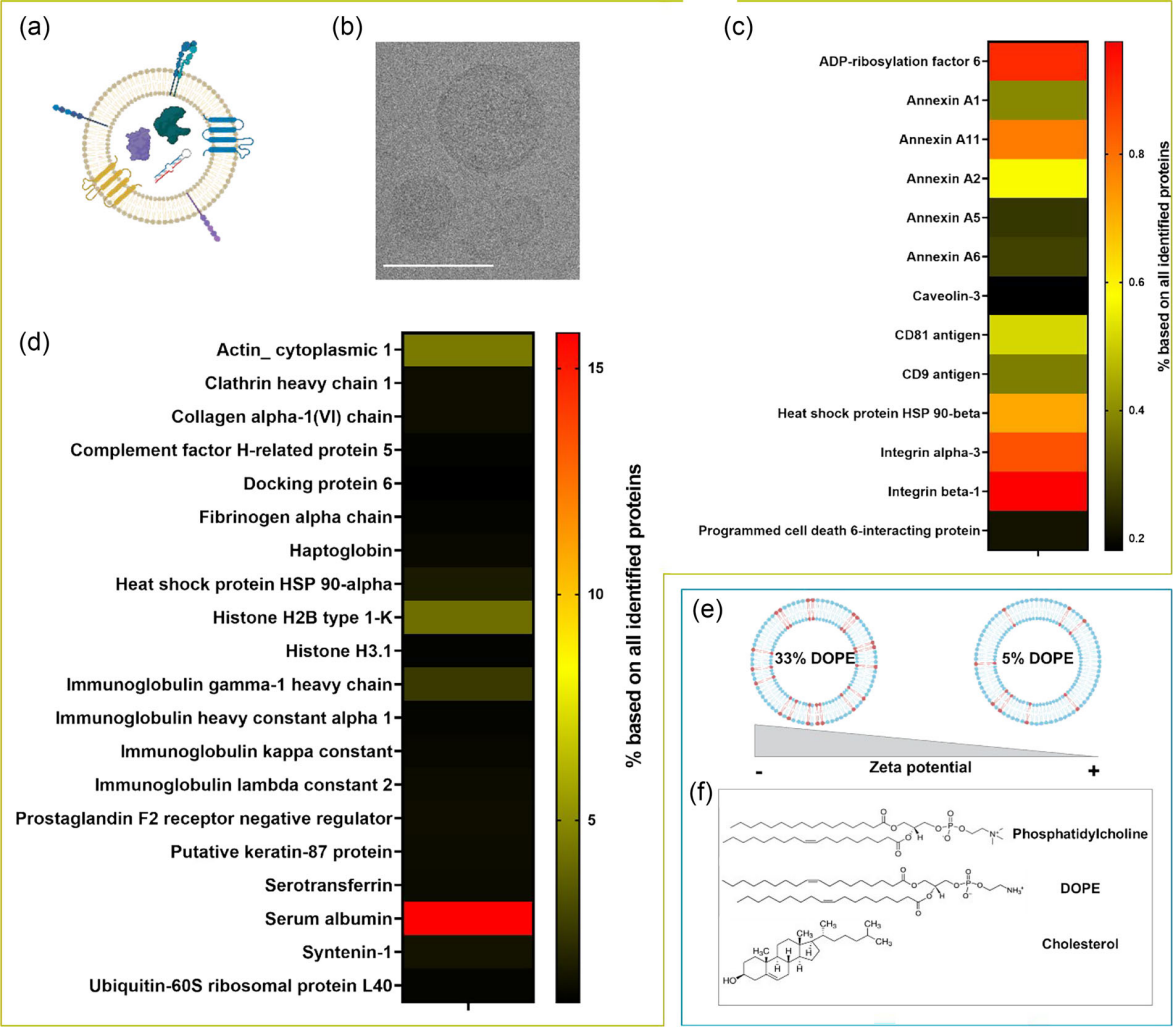
Characterization of liposomes and HCT 116‐derived EVs. (a) Schematic depiction of EV. (b) Cryo‐TEM image of EV preparation. Scale bar: 100 nm. (c) EV marker proteins in EV preparation identified by MS analysis. Accession numbers of the proteins are found in SI Table 1. (d) TOP 20 proteins of proteomic analysis of EV preparation. Accession numbers of the proteins are found in SI Excel Sheet “AccessionNumber_20MostAbundantProteins.xlsx.” (e) Schematic depiction of relation of liposomal DOPE content and zeta potential. (f) Lipid components of liposomes.

Figure 2 depicts the molecular differences between EVs (Figure 2a‐d) and liposomes (Figure 2e‐f). As shown in Figure 2a, EVs contain transmembrane proteins, which are inserted into the lipid bilayer and together with the glycocalyx alter the EV surface. Additionally, they contain soluble proteins and metabolites in the aqueous lumen (van Niel et al., 2018). The cryo‐TEM image of the EV preparation depicted in Figure 2b showed round vesicular structures with a diameter between 50 and 100 nm, which fits well with typical EV sizes. The characteristic lipid bilayer of the EVs is even visible in this cryo‐TEM image. The un‐cropped image is provided in SI Figure 1a. Proteomic analysis of nascent EVs depicted in Figure 2c demonstrated the presence of common EV markers according to MISEV guidelines (Théry et al., 2018). The EV markers were found with low abundancies and were therefore not found among the TOP 20 identified proteins which are depicted in Figure 2d. The tetraspanins CD81 and CD9 were identified. Furthermore, integrins α and β, different annexins, caveolin, ADP‐ribosylation factor 6 (ARF6), HSP90‐β (HSP90AB1) and ALIX (Programmed cell death 6‐interacting protein) were detected in the isolated EVs. Calnexin which is a marker for endoplasmic reticulum contamination was not detected (Figure 2c and SI Table 1). We further analyzed the EV diameter with DLS and NTA and determined the size distribution of vesicles imaged with Cryo‐TEM. Additionally, we measured the zeta potential of an EV sample (Table 1 and SI Figure 2). The mean hydrodynamic diameter determined by NTA was 113 nm, which was in good accordance with the mean diameter observed in cryo‐TEM images, 91 nm. As visible by cryo‐TEM, the EV sample is a polydisperse vesicle solution and there were small vesicles observed between 30 and 60 nm in diameter. Larger vesicles were also captured by the NTA measurement as the histogram showed a shoulder reaching towards diameters of 500 nm (SI Figure 4). DLS values were not given for the EVs, as the sample was too polydisperse for this measurement technique (Caputo et al., 2021). The zeta potential in absence of a protein corona was near neutral, which is in line with previous reports (Wolf et al., 2022).

**TABLE 1:** Physical characterization of EVs and liposomes.

	D_DLS_ [nm]	PDI_DLS_	D_NTA_ [nm]	D_TEM_ [nm]	ζ‐potential [mV]
HCT 116 EVs	–	–	113 ± 54	91 ± 109	−3 ± 0.4
33%DOPE	189 ± 1	0.125 ± 0.04	–	–	−21 ± 0.2
5%DOPE	206 ± 4	0.153 ± 0.03	–	–	−12 ± 0.3

*Note*: The hydrodynamic diameter was determined by DLS and NTA, both measured in PBS at 25°C. For HCT 116 EVs, no DLS and PDI measurements were given as the population is too polydisperse. Additionally, the physical diameter was determined by analyzing cryo‐TEM images. Means and standard deviations are shown. The zeta potential was measured at 20°C in 1 mM KCl. Means and standard deviations are shown.

In contrast to EVs, liposomes are composed of a lipid bilayer that encloses an aqueous lumen but do not contain any additional proteins or metabolites (Figure 2e). Here, we used liposomes prepared with phosphatidylcholine, cholesterol, and varying amounts of DOPE to vary the zeta potential (Figure 2f). The liposomes were prepared by thin film hydration followed by extrusion to have a diameter around 200 nm as previously reported (Gai et al., 2020). The DLS measurement of the liposomes gave a diameter of 189 and 206 nm for the 33% and 5% DOPE liposomes (Table 1) and a narrow size distribution was observed (SI Figure 3). Depending on the DOPE amount, liposomes had a negative zeta potential of −20.6 and −11.8 for high and low DOPE liposomes, respectively (Table 1). In neutral pH, DOPE carries a negatively charged head group. Therefore, the more DOPE is incorporated into the lipid membrane the more negative the zeta potential will be. Additionally, DOPE carries a NH_2_ group which is used for fluorescent labelling. At a DOPE content of 5% the fluorescence labelling is week. At a DOPE content of 33% a higher fluorescence is achieved as more coupling moieties are available. Reducing the DOPE content further would shift the zeta potential towards an even more neutral zeta potential which would be more like that of EVs. However, this would reduce the fluorescence intensity too much. Therefore, we decided to use one liposome that was highly fluorescence but had a more negative zeta potential then EVs and one liposome that was weekly fluorescence but had a more similar zeta potential compared to EVs.

**FIGURE 3:**
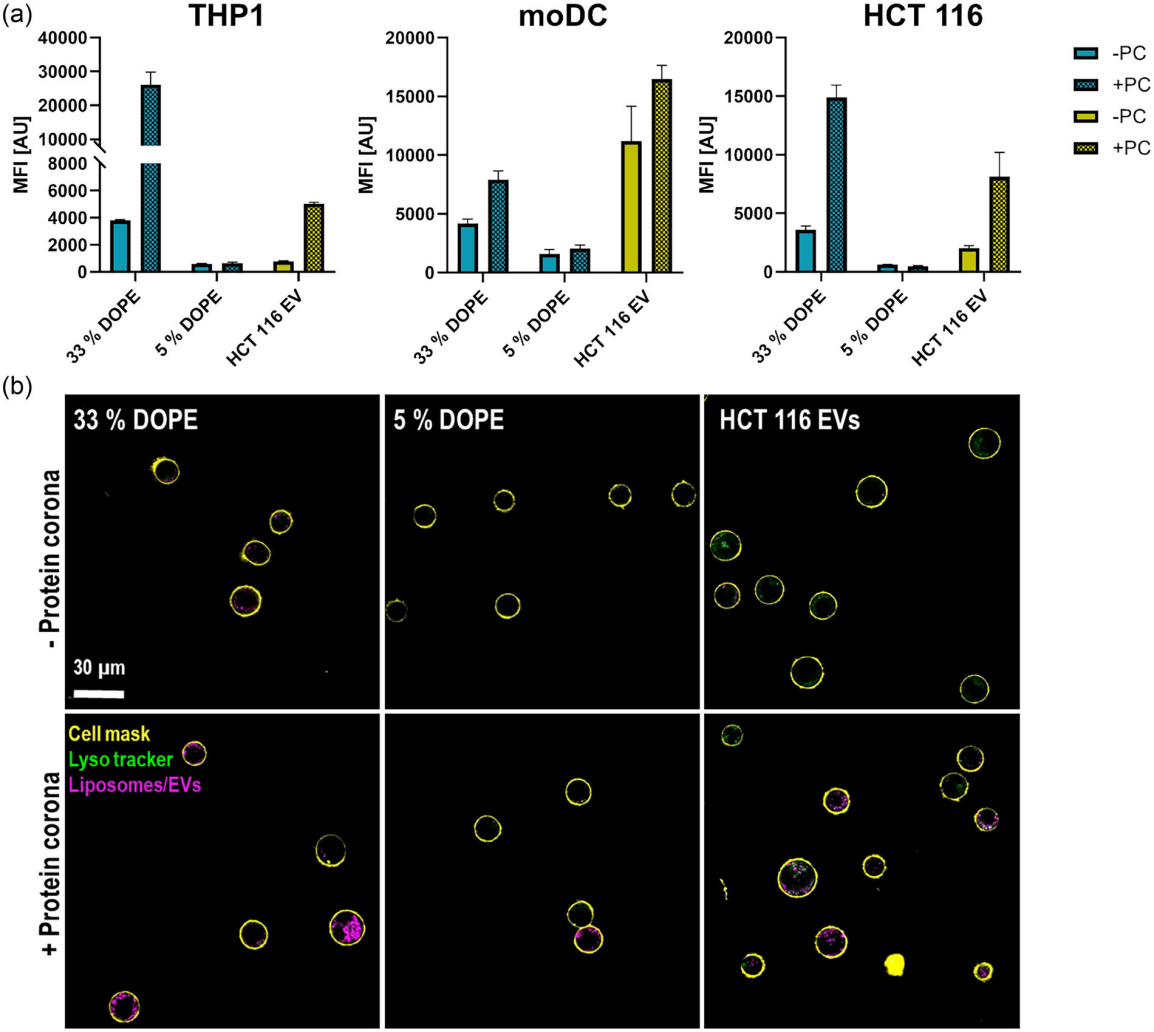
Uptake of liposomes and EVs with and without protein corona. (a) Flow cytometric analyses of particle uptake into THP1 cells, moDCs and HCT 116 cells after 16 h. The MFI values of untreated cells were 182 ± 2.9 AU for THP1 cells, 5243.3 ± 110 AU for moDCs and 238 ± 11.6 AU for HCT 116 cells. The MFI values of untreated were subtracted as background signal from all other values. Means and standard deviations of median fluorescence intensities are shown (*n* = 3). (b) Confocal laser scanning microscopy images of THP1 cells incubated with liposomes or EVs in presence (lower) and absence (upper) of a protein corona for 16 h. The cell membrane is coloured in yellow, the lysosome is pseudocoloured in green and liposomes/EVs are pseudocoloured in magenta. Scale bar: 30 µm.

As it is well known for synthetic nanoparticles that a protein corona can alter cell uptake, we investigated the influence of a protein corona on uptake of EVs (Prawatborisut et al., 2022; Ritz et al., 2015; Schöttler et al., 2016). Depending on the particle surface and cell type, pre‐adsorption of a protein corona can result in a reduced or enhanced uptake of nanoparticles by the recipient cells (Berrecoso et al., 2020). Here, we tested the influence on uptake of EVs that expose on their surface a protein corona or not in human phagocytes (THP1, moDCs) and as a non‐phagocytic cell type, the cells the EVs were derived from (HCT 116), thus simulating the uptake by neighbouring or distant tumour cells (e.g., metastases). To this end, we incubated 10 µg mL^−1^ of Cy5‐labelled liposomes or EVs with human blood plasma at 37°C for 1 h and subsequently isolated them by centrifugation. The last pellet resulting from centrifugation was re‐suspended in serum‐free cell culture medium to perform the cell uptake under serum‐free conditions for samples with and without pre‐adsorbed protein corona. This was done to avoid the formation of a serum protein corona on plain EVs or exchange of proteins from the pre‐formed human plasma protein corona with serum proteins from fetal calf serum (FCS). In our study, we focus on the difference between particles with and without a pre‐formed protein corona. Thus, our results inform about the contribution of corona proteins and neglect the eventual effect of additional free plasma proteins. However, Yang et al. demonstrate that free serum proteins can also contribute to the differential uptake in presence of a protein corona as they for example, compete with particles for receptor binding. This effect might also play a role in EV uptake in presence of a human plasma protein corona. The uptake of EVs and liposomes with and without a protein corona was analyzed by flow cytometry (Figure 3). As shown in SI Figure 5 the vesicles differed in fluorescence intensity due to inequalities during the labelling process which is inherent to the amounts of possibly reactive side groups. Therefore, mean fluorescence intensity (MFI) values cannot be directly compared between vesicle types. In addition, the recovery efficiencies of vesicles after incubation with plasma (+PC) and PBS (‐PC) differ. To account for particle loss after protein adsorption in plasma, MFI values were calibrated according to fluorescence intensity of + and –PC samples. In contrast to many publications before on nanoparticles where the protein corona had an anti‐uptake effect, pre‐adsorption of a protein corona increased MFI values of high DOPE liposomes (33% DOPE) and EVs in all tested cell types (Figure 3a). For 5% DOPE liposomes no major differences were detected with or without PC. For all cell lines, the percentage of positive cells was near 100% when incubating with high DOPE liposomes suggesting that all cells have taken up particles (SI Figure 6). Differences between different nanoparticle formulations could be observed when analyzing the median fluorescence intensities indicating the amounts of nanocarriers taken up by cells. For 5% DOPE liposomes, the vesicles without protein corona showed near 100% uptake in THP1 and HCT cells, but almost no uptake in moDCs. In contrast, the 5% DOPE liposomes with protein corona showed uptake in around 25% of THP1 and HCT 116 cells (SI Figure 6). Due to loss of liposomes and EVs during protein corona formation we adjusted the MFI values according to the different fluorescence intensities. Thereby, the inverted trend in MFI values in comparison to the percentage of particle‐positive cells can be explained. This shows that the reduced percentage of particle‐positive cells is due to loss of liposomes during protein corona formation and not due to reduced uptake in presence of a protein corona. For EV samples, the increased MFI values in presence of a protein corona is reflected by the percentage of positive cells in THP1 and HCT 116 cells. In moDCs + and—PC showed near 100% uptake.

To validate vesicle uptake in THP1 cells, we additionally obtained cLSM images of cells incubated with Cy5‐labelled liposomes or EVs (Figure 3b). To account for differences in fluorescence intensity of + and –PC samples, we used fluorescence calibration to determine the number of vesicles added to the cells. The microscopy images are in good accordance with the results from flow cytometry studies. For all vesicles, a higher uptake is observed after the adsorption of a protein corona. Furthermore, vesicle signals were detected intracellularly confirming uptake into the cell.

The ATP measurements were done in serum‐free conditions. This was done to match the cell uptake conditions. After 16 h of serum‐free cultivation the cells may have entered starvation mode which can reduce the cell viability and thus ATP content. That makes them potentially more susceptible for additional stresses like particle uptake.

Uptake of vesicles independent of pre‐adsorption of plasma proteins decreased ATP levels of THP1 cells to 60–70% of untreated cells (SI Figure 7). The metabolic activity of the cells was decreased by the interplay of serum‐starvation and particle uptake. However, no cells with abnormal morphology were observed under the microscope. Therefore, the cells possibly decrease metabolic activity upon particle uptake but do not enter cell death.

HCT cell semmed to be less affected by the serum‐free condition. Here, no decrease in ATP levels was observed after incubation with the liposomes or EVs.

EVs were taken up by the phagocytic THP1 cells and moDCs, as well as by the non‐phagocytic HCT 116 cell line. This might also reflect the fact that TEX exert functional changes in many different cell types ranging from metastatic tumour cells to immune and stromal cells in the tumour environment (Dai et al., 2020). Furthermore, EV uptake was increased upon protein corona adsorption by the two phagocytic cell types. This suggests that an opsonin‐rich protein corona was formed probably resulting in rapid blood clearance if these would be used in vivo. Additionally, EV uptake in HCT 116 cells was also enhanced upon protein corona adsorption. In contrast to phagocytic uptake of EVs, this would be advantageous for a delivery strategy that utilizes tumour‐homing of EVs to the cells they originated from. In the context of tumour progression, uptake of TEX by tumour cells of origin is a mechanism to distribute chemo resistance and oncogenic traits even to distant tumour metastases (Asare‐Werehene et al., 2020; Crow et al., 2017; Paskeh et al., 2022). Here, enhanced uptake of TEX by the tumour cells in the presence of a protein corona can be a mechanism to enhance uptake by metastatic tumour cells in contrast to neighbouring tumour cells.

Previous studies have demonstrated adsorption of a protein corona on EVs increases their functionality. EVs with a protein corona showed a pronounced effect on target cells, for example, resulting in increased interferon secretion, angiogenesis, or T cell inhibition (Tóth et al., 2021; Wolf et al., 2022). Facilitating an increased uptake of EVs could explain the enhanced biological activity in presence of a protein corona. Moreover, the fact that we observed an enhanced uptake of EVs with protein corona in primary moDCs concur with the finding that EVs with protein corona facilitate enhanced interferon secretion in monocyte‐derived dendritic cells (Tóth et al., 2021). In fact, the increased uptake could be the mode of action to enhance EV functionality.

In order to understand the enhanced uptake of liposomes and EVs in presence of a protein corona, we used LC‐MS to identify the protein corona composition. The isolation protocol applied in this study separates vesicles based on their size regardless of displayed surface markers. Therefore, the resulting samples contain a mixture of different EV subtypes such as exosomes, microvesicles and apoptotic bodies that are within a size range of approximately 50–200 nm. In conclusion, the corona studied here was a mixture of different coronas forming on these different vesicles. However, the mixed population examined here is of relevance for research and clinical application as SEC is among the most popular EV isolation methods.

**FIGURE 4:**
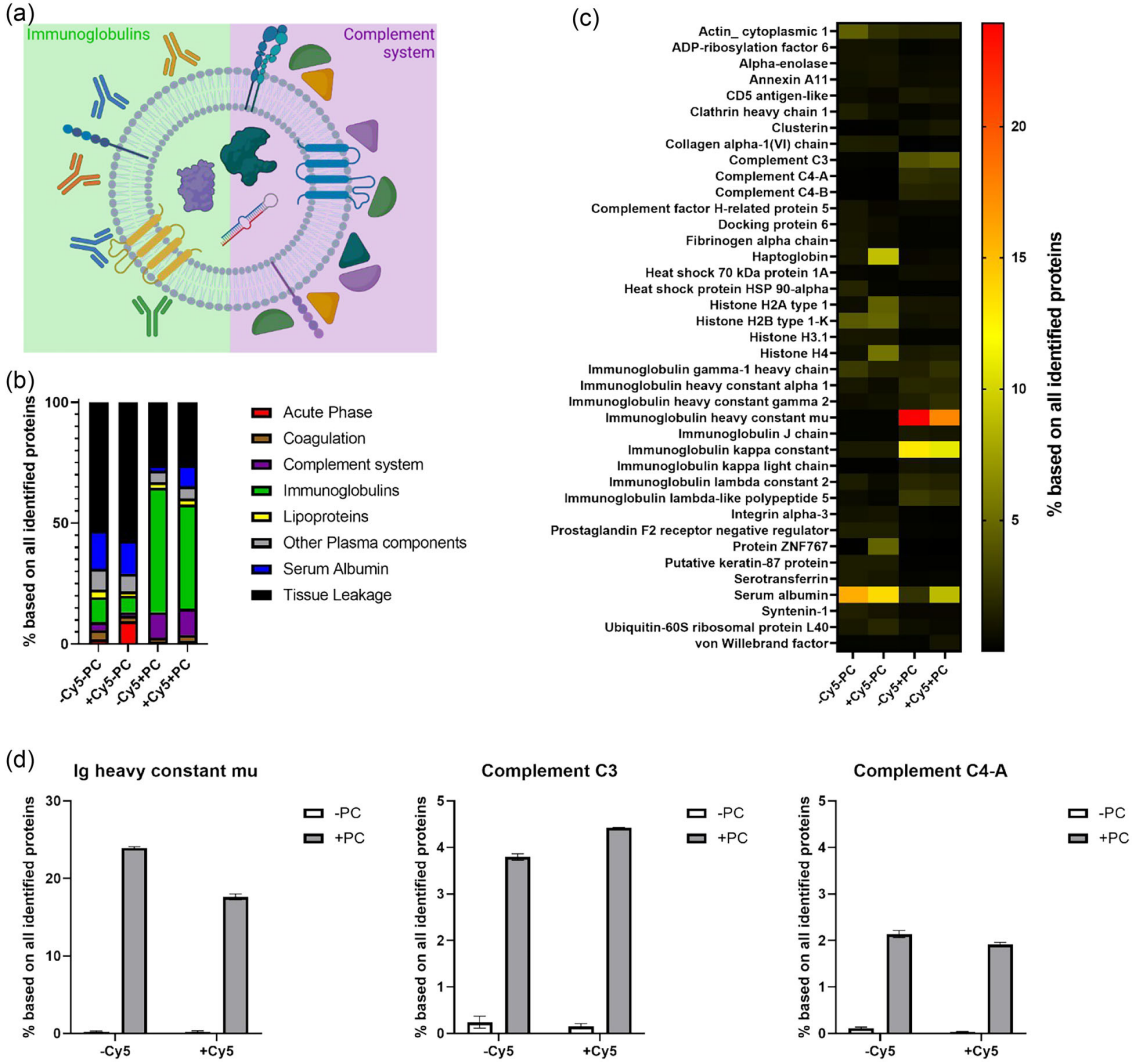
Proteomic analysis of EV and protein corona composition. (a) Graphic depiction of enrichment of immunoglobulins and complement system proteins in EV protein corona. (b) Assignment of proteins to different biofunctional classes. (c) LC‐MS revealed the top 20 hard protein corona proteins of EVs with and without Cy5‐labelling. Accession numbers of the proteins are in the SI Excel Sheet “AccessionNumber_20MostAbundantProteins.xlsx.” (d) Enrichment of Ig heavy constant mu, complement C3 and complement C3 in protein corona on EVs with and without Cy5‐labelling. Mean and standard deviation of three technical replicates are shown.

Since the fluorescent Cy5‐labelling necessary for cell uptake analysis alters liposomes’ and EVs’ surfaces, it could influence the protein corona formation around the vesicles. Analyzing the corona composition of unlabelled and Cy5‐labelled EVs and liposomes did not show major differences (Figure 4 and SI Figure 8). The zeta potential of liposomes and EVs after adsorption of a protein corona was ranging between −26.2 and −17.4 mV as depicted in SI Figure 9, which is within the expected range and suggests adsorption of a protein corona (Wolf et al., 2022).

Prior to MS analyses, the protein corona of liposomes was detached as this is the standard protocol for preparation of a liposome protein corona and ensures comparability to former publications. This was not the case for EVs, where we analyzed a sample with and without previous adsorption of a protein corona in parallel as this also gives us the functional proteins of the EVs. Also separation of adhered proteins from the protein corona and adhering or partially integrated proteins into the EV membrane could not be easily separated by a physical process like washing and centrifugation. Proteins that were only enriched in the sample with adsorbed protein corona were accounted as corona proteins from the blood plasma. All identified proteins and their relative amounts are listed in a separate file (see Supporting Information). Unlike previous studies, we used untreated plasma that was not depleted from plasma‐derived EVs or lipid particles before protein corona formation. That was done to ensure that the plasma has a more original composition but also bears the risk of contamination of the MS results with plasma‐derived EV and lipid particle proteins. To estimate the degree of contamination by plasma‐derived EVs and lipid particles in our EV protein corona, we prepared process controls. That is a sample that undergoes the complete protein corona adsorption and isolation protocol but do not contain additional EVs. The plasma process control had a protein concentration of 0.0022 mg/mL and the LC‐MS analysis revealed only 8 proteins in total (SI Table 2). This is close to the background of an empty sample. In comparison, the EV protein corona sample had a protein concentration of 0.195 mg/mL and 225 proteins could be identified (see SI Excel Sheet “LC_MS_data_EVCorona.xlsx”). Therefore, we considered the contribution of plasma‐derived EVs and lipid particles as neglectable. The EV protein corona was mainly enriched with immunoglobulins and complement system proteins (Figure 4a,b). The biofunctional class of tissue leakage proteins, as well as serum albumin were decreased upon adsorption of a protein corona (Figure 4b). In particular, complement C3 and C4 were found. For immunoglobulins, immunoglobulin heavy constant µ and κ constant were the most abundant proteins and we also identified immunoglobulin heavy constant γ 2 (Figure 4c). One of the most enriched proteins was immunoglobulin heavy constant µ which increased from approximately 0.3% to 24% of all identified proteins after adsorption of a protein corona. A similar trend was observed for complement C3 and C4‐A, which increased from 0.1% to 4% and 0.1% to 2% of all identified proteins, respectively (Figure 4d). A strong increase was also detected for immunoglobulin κ constant and complement C4‐B (SI Figure 10). Next, we compared the most abundant protein corona proteins identified by us to major EV protein corona components identified by Tóth et al. (2021). Immunoglobulin heavy constant γ 2, complement C3 and C4‐B and fibrinogen α‐chain identified by us are also proposed as major EV protein corona proteins by Toth et al. Additionally, they proposed ApoA1, ApoB, ApoC3, ApoE and immunoglobulin heavy constant γ 4. ApoA1 and ApoE were also found with lower abundancies in our samples. Furthermore, we identified clusterin among the top EV corona proteins representing a member of the apolipoprotein family (Figure 4c). Comparing the protein corona of unlabelled and Cy5‐labelled EVs showed a similar protein composition. The only difference for the Cy5‐labelled EV's protein corona was the higher prevalence of serum albumin and less immunoglobulin heavy constant µ, but still these proteins were in the top 20 list of proteins found. This is well within the differences of the semi‐quantitative assessment of protein quantification in an un‐labelled liquid chromatography—mass spectrometry (LC‐MS) setup.

We also compared the most abundant EV corona proteins to liposome corona proteins (SI Figure 8). Despite the difference in zeta potential, the protein corona of high‐ and low‐DOPE liposomes showed very similar trends as both protein coronas were enriched with apolipoproteins, immunoglobulins, and serum albumin. In contrast to the EV protein corona, complement proteins were not highly abundant. The EV protein corona was generally more complex but was less enriched for apolipoproteins and serum albumin. Comparing the protein corona of unlabelled and Cy5‐labelled liposomes showed minor differences for the 5% DOPE liposomes. In the protein corona of the labelled liposomes less serum albumin, but more tissue leakage proteins and lipoproteins were found compared to the unlabelled 5% DOPE liposomes. Also this we would consider to be within the limit of assessment in a proteomics LC‐MS setup. The liposomes used here, have a more negative zeta potential compared to the EVs. This could rise the question, if the difference in protein corona observed between liposomes and EVs is due to the difference in zeta potential. However, the protein corona of the two differently charged liposomes was almost identical. Therefore, we conclude that the zeta potential of the liposomes plays a minor role for their protein corona composition and the difference in protein corona composition between EVs and liposomes is not mainly caused by the difference in zeta potential.

As observed for liposomes before, the EVs used in this study showed an enhanced uptake in phagocytic cell types after protein corona adsorption. We therefore investigated if the high amount of immunoglobulins representing known opsonins would explain the higher uptake. In the 33% DOPE liposome protein corona, we also found immunoglobulins but fewer complement proteins. Instead, apolipoproteins and serum albumin were enriched, which are known as dysopsonins. However, the dysopsonizing proteins could not ameliorate the opsonizing effect of immunoglobulins. Overall, the protein coronas of 33% DOPE liposomes and EVs had opsonizing properties as cell uptake into phagocytes was increased upon protein corona adsorption.

Comparing our results to Mateos‐Maroto et al., also stresses the importance of investigating human plasma‐derived protein coronas for targeting of human cell lines when developing drug delivery vehicles (Mateos‐Maroto et al., 2023). In contrast to our results, they found that an FBS‐derived protein corona decreased the uptake of liposomes in murine macrophage cells. For liposomes, it has been known for decades that rapid blood‐clearance is a major problem for clinical applicability and surface modifications are used to prolong blood circulation times (Shen et al., 2018). This is in good accordance with our finding, that the protein corona on non‐functionalized liposomes enhances uptake in monocytes. Furthermore, this raises the question if EVs would also need stealth modifications to reach their full delivery potential.

**FIGURE 5:**
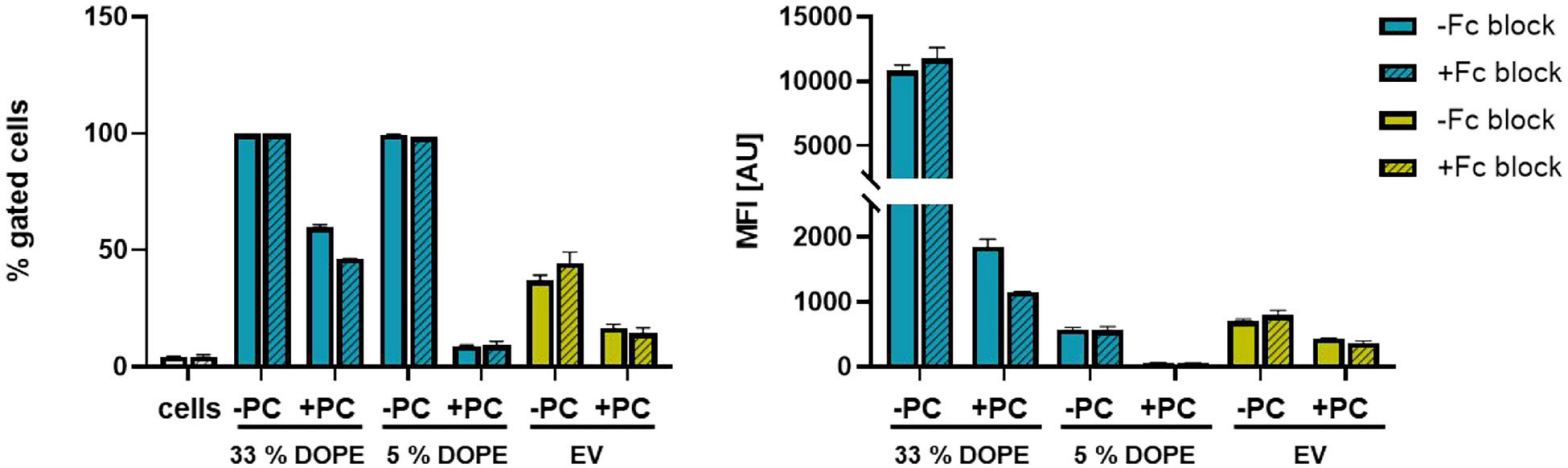
Binding of liposomes and EVs in absence and presence of Fc‐receptor blocking. Flow cytometric analysis of vesicle binding to THP1 cells after 40 min incubation at 4°C. The MFI value of untreated cells was 234 ± 2.1 AU. This value was subtracted as background signal from all other values. Means and standard deviations of % gated cells and MFI are shown (*n* = 3).

As we found immunoglobulins enriched in liposome and EV protein coronas, we intended to test if the enhanced uptake into THP1 cells is Fc‐receptor mediated. Fc‐receptors are expressed on monocytes and macrophages and are one of the main receptors responsible for the clearance of nanoparticles (Gustafson et al., 2015). The receptor recognizes the Fc‐region of IgGs. Therefore, adsorption of IgGs on nanoparticles can lead to adherence to the cell and ultimately phagocytosis (Aderem & Underhill, 1999). We therefore blocked cell surface Fc‐receptors. Subsequently, THP1 cells were incubated with vesicles at 4°C to avoid turn‐over of Fc‐receptors or internalization of vesicles (Figure 5). Interestingly, the mere cell surface binding of liposomes and EVs was higher in absence of a protein corona. In contrast, internalization of 33% DOPE liposome and EVs was higher in presence of a protein corona (compare Figure 3 and Figure 5). This suggests that binding to the cell surface (at 4°C) and uptake into the cells (at 37°C) is influenced by the presence of proteins differently. This can be explained as binding to the cell surface is determined by the amount of receptors on the cell surface, while uptake is a distinct process and a highly abundant or strongly binding receptor may only be sluggishly taken up while a receptor expressed in low numbers may be rapidly taken up. This also gives a hint that two different uptake mechanisms are responsible for the internalization of liposomes and EVs in the presence and absence of a protein corona. Indeed, it has been previously reported that the presence and composition of the protein corona can influence the uptake mechanisms of nanoparticles at 37°C (Digiacomo et al., 2017; Francia et al., 2019). When Fc‐receptors were blocked in the absence of a protein corona, we did not observe differences in the binding of vesicles (at 4°C) as expected. In the presence of a protein corona, we observed a decreased binding of 33% DOPE liposomes when Fc‐receptors were blocked, determining that for liposomes immunoglobulins in the protein corona contribute to enhanced uptake. In contrast to this, for EVs we did not observe a reduction of binding in the presence of Fc‐receptor block. Therefore, we conclude, that for EVs, Fc‐receptors seemed not to be prominently involved in cell binding despite the presence of IgGs in the protein corona. This is also a clear difference with the findings for the liposomes. This is an interesting finding as it hints towards a hitherto undetermined receptor responsible for uptake of protein coated EVs. For this effect a wide range of additional receptors could be responsible. In addition to Fc‐receptors, monocytes exhibit mannose receptors, scavenger receptors and complement receptors that are capable of recognizing foreign materials (Gustafson et al., 2015; Sharma et al., 2017). They can also recognize phosphatidylserine in the outer leaflet as found in apoptotic cells (Aderem & Underhill, 1999). This could also be a possible mechanism for the recognition of EVs as they often expose phosphatidylserine on their surface (Sharma et al., 2017).

## 4 CONCLUSION

The rising field of tumour vaccination also brings tumour‐derived exosomes into focus as vaccination agent. Here, a strong uptake into immune cells such as monocytes is rather favourable as it is expected to facilitate immune response. In fact, enhanced uptake into monocytes, a dendritic cell precursor, could be an advantage for the development of effective anti‐tumour vaccines. Therefore, our observations provide important insides into the role of a protein corona for successful uptake in moDCs in the context of immune modulation and tumour‐vaccination.

In this study, we demonstrate that liposome and EV uptake into phagocytic cells is enhanced in the presence of a protein corona. For 33% DOPE liposomes immunoglobulins were found in the corona as well, presumably mediating at least partially the opsonizing effect by enhanced surface binding. For EVs, this cannot be explained by the enrichment of immunoglobulins as opsonizing proteins, but rather other protein corona components would need to take up this function or the depletion of dysopsonizing proteins like apolipoproteins is responsible for this effect. Our data provide valuable insights into the mode of action of enhanced EV functionality in presence of a protein corona. Furthermore, our results suggest that for successful vaccination using tumour‐derived EVs, the protein corona plays an enhancing role. Generally, this supports the paradigm shift towards corona proteins being an integral part of EV functionality, which is an important step not only for EV research but also for the development of EVs as a drug delivery platform.

## AUTHOR CONTRIBUTION

**Laura Dietz**: Data Curation; formal analysis; investigation; validation; visualization; writing—original draft; writing—review and editing. **Jennifer Oberländer**: Data Curation; formal analysis; validation; visualization; writing—original draft; writing—review and editing. **Ana Mateos‐Maroto** Data Curation; formal analysis. **Jenny Schunke**: Data Curation; formal analysis. **Michael Fichter**: Formal analysis. **Eva‐Maria Krämer‐Albers**: Supervision; project administration. **Katharina Landfester**: Funding Acquisition; project administration; supervision. **Volker Mailänder**: Funding Acquisition; project administration; supervision.

## CONFLICT OF INTEREST STATEMENT

The authors declare no conflicts of interest.

## Supporting information

Supporting Information

Supporting Information

Supporting Information

## Data Availability

Data is available on request from the authors.
