# Supplementary material for: Uptake of extracellular vesicles into immune cells is enhanced by the protein corona
Source: J Extracell Vesicles. 2023 Dec 20;12(12):e12399. doi: 10.1002/jev2.12399 (PMC10733601; doi:10.1002/jev2.12399)
Supplement: Supplementary file 1 — Supporting Information [file JEV2-12-e12399-s001.docx]

Supplementary information


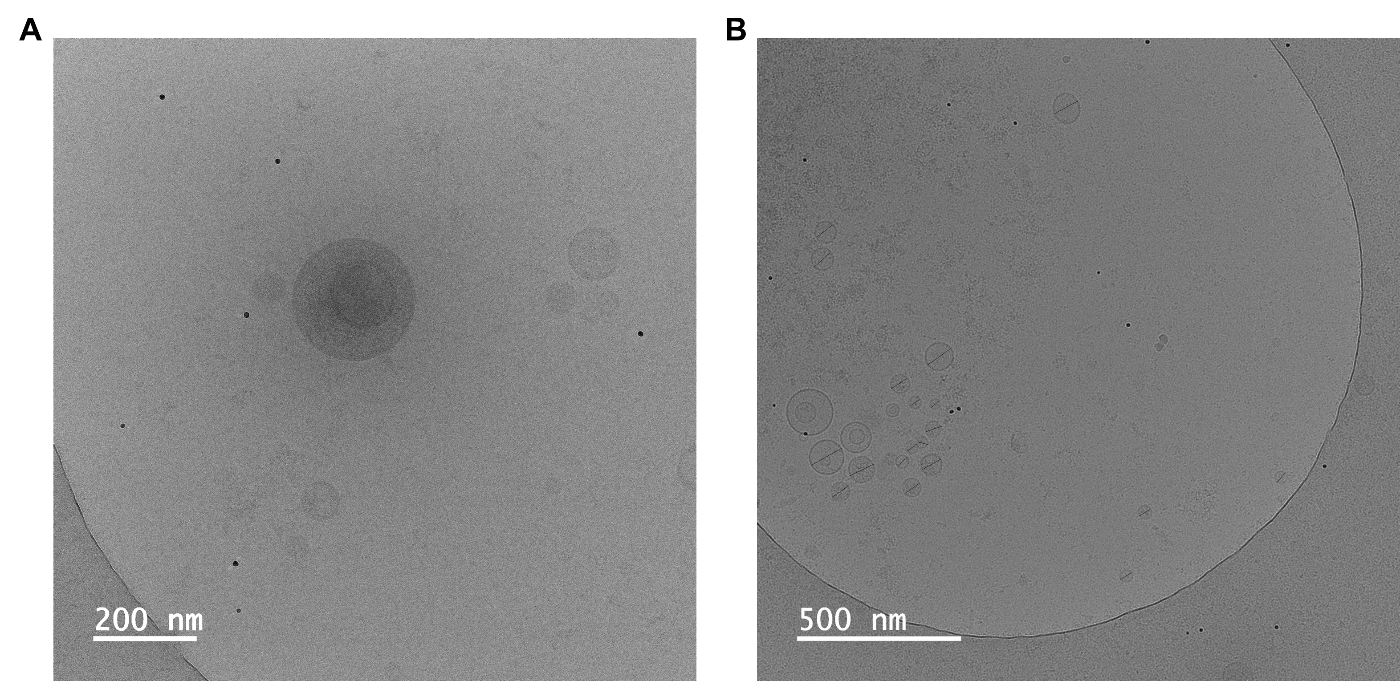


**SI figure 1:** Cryo-TEM images of EV preparation. (A) The un-cropped image of the image shown in B. (B) Example image to showcase, how diameter measurments were done. The measured diameter distances are indicated in black.


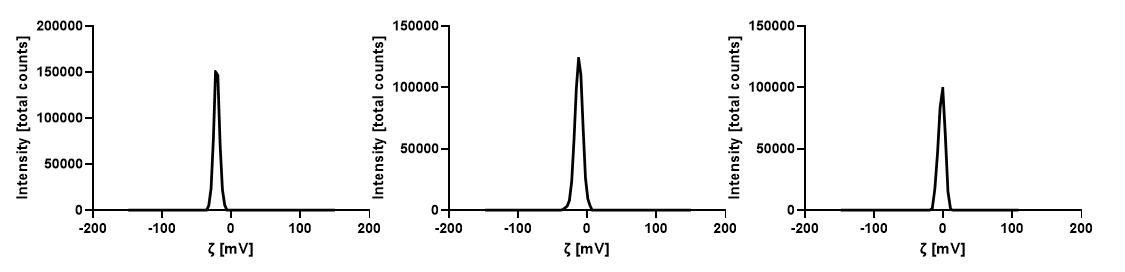


**SI figure 2:** Intensity measurement of zeta potential for (A) 33 % DOPE liposomes; (B) 5% DOPE liposomes and (C) HCT 116-derived EVs.


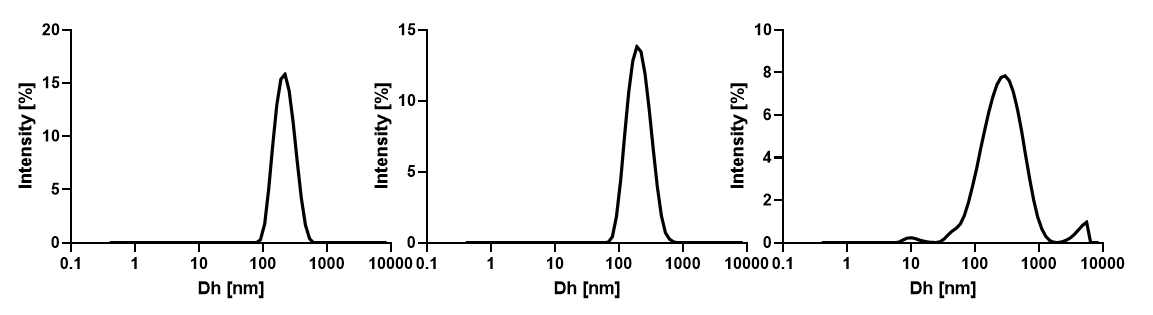


**SI figure 3:** Intensity measurement of hydrodynamic diameter measured for (A) 33% DOPE liposomes and (B) 5% DOPE liposomes.


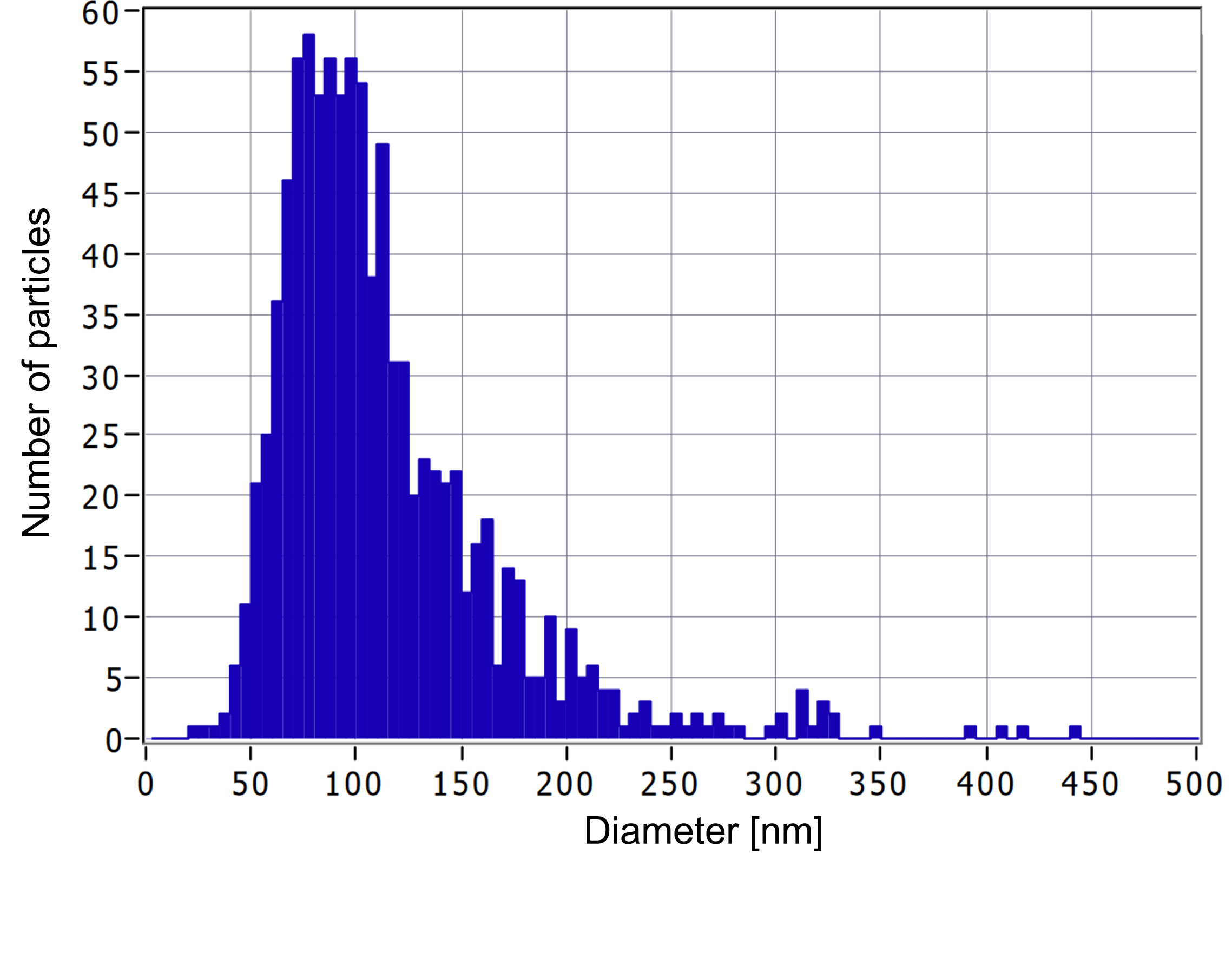


**SI figure 4:** Histogram of particle size distribution measured by NTA.


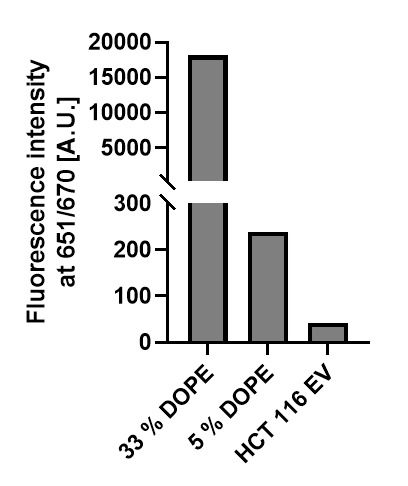


**SI figure 5:** Cy5 fluorescence intensity of liposomes and EVs after staining. Fluorophore was excited at 651 nm ± 5 nm and detected at 670 nm ± 5 nm.


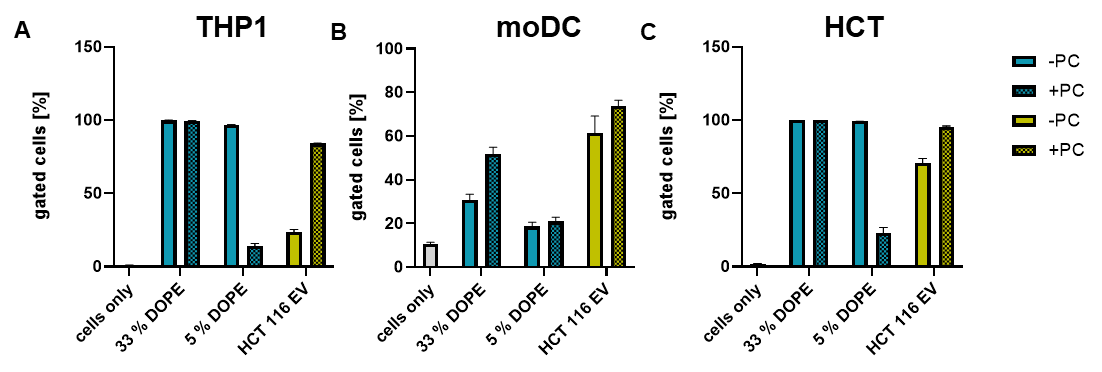


**SI figure 6:** Uptake of liposomes and EVs with and without protein corona. Flow cytometric analyses of particle uptake into THP1 (A), moDC (B) and HCT 116 (C) cells after 16h. Means and standard deviations of % gated cells are shown (n=3).


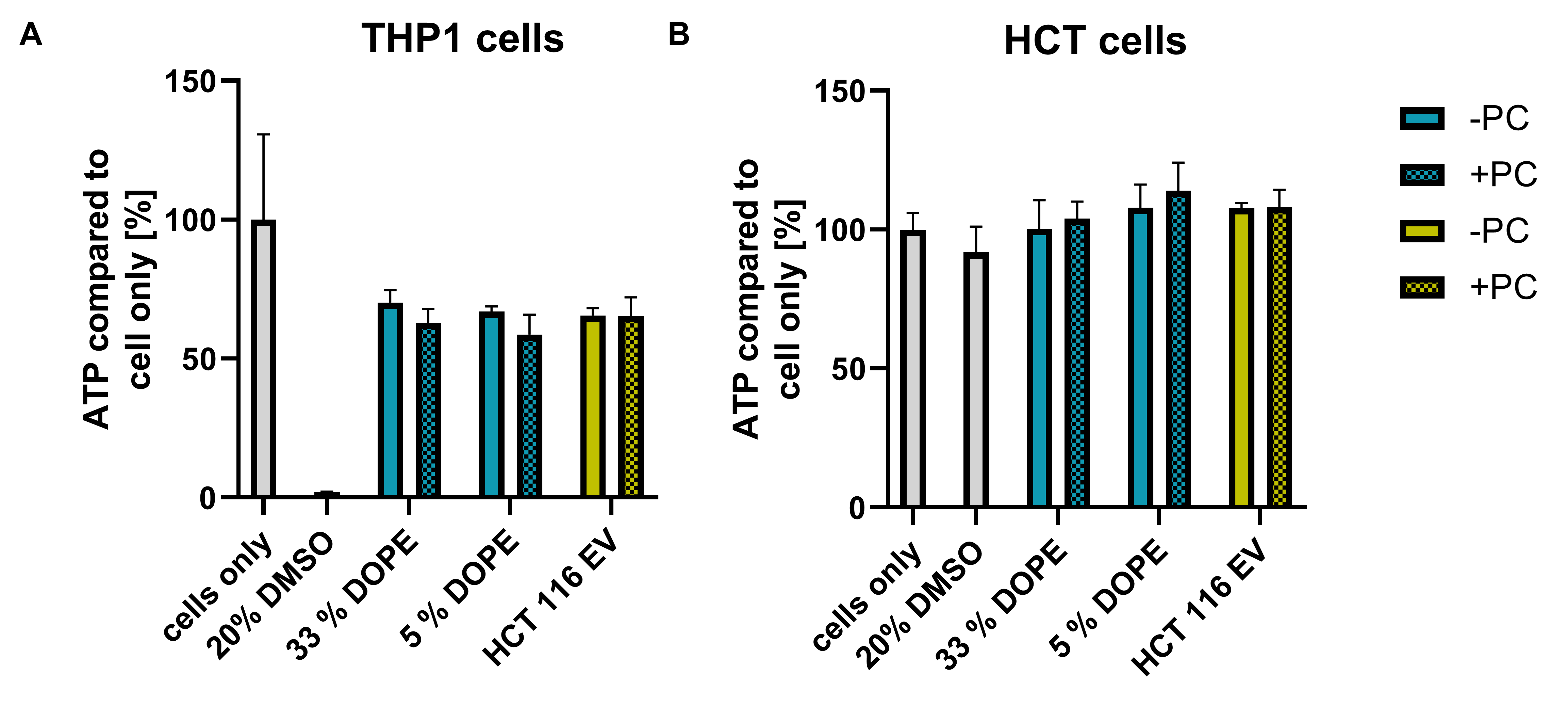


***SI figure 7:*** *ATP content of cells treated with liposomes or EVs with and without protein corona. THP1 (A) or HCT 116 (B) cells were incubated with particles or 20% DMSO (positive control) for 16 h. Means and standard deviations are shown (n=3).*


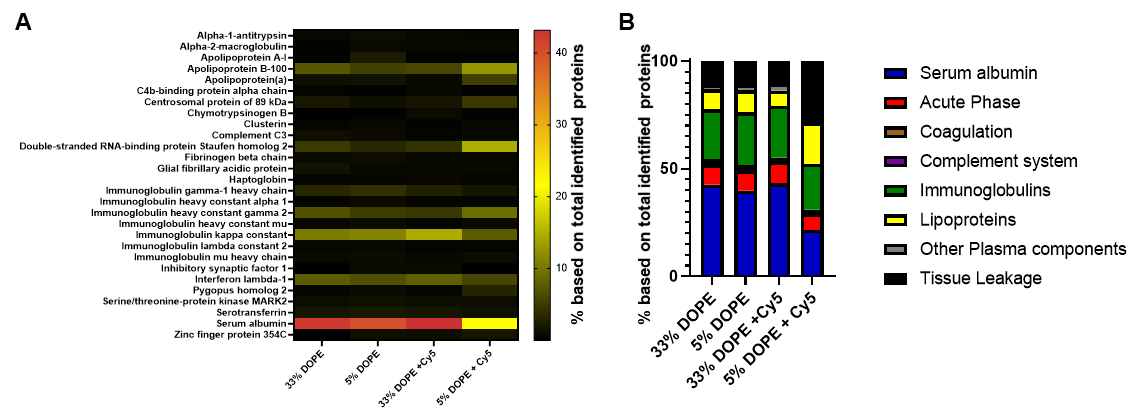


**SI figure 8:** Analysis of proteins found in liposome hard protein corona. (A) LC-MS revealed the top 20 hard protein corona proteins of 33% and 5% DOPE liposomes with and without Cy5-labeling. Accession numbers of the proteins are in the SI Excel Sheet “AccessionNumber_20MostAbundantProteins.xlsx”. (B) Assignment of proteins to different biofunctional classes.


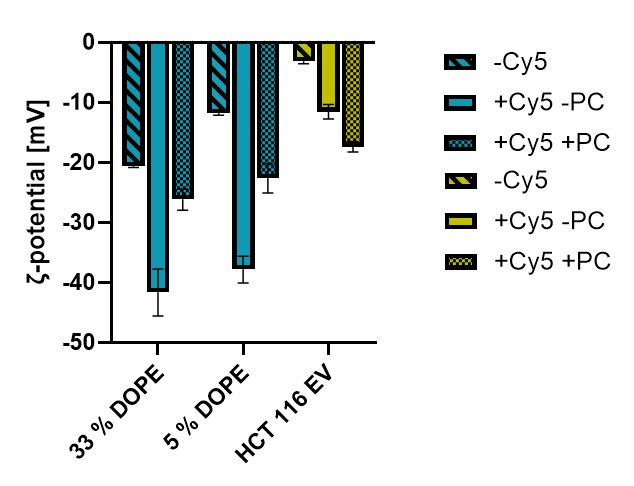


***SI figure 9:*** *Zeta potential of vesicles without Cy5-labeling and with Cy5-labeling in absence or presence of a protein corona. Measurement was performed at 20 °C in 1 mM KCL. Means and standard deviations are shown (n=3).*


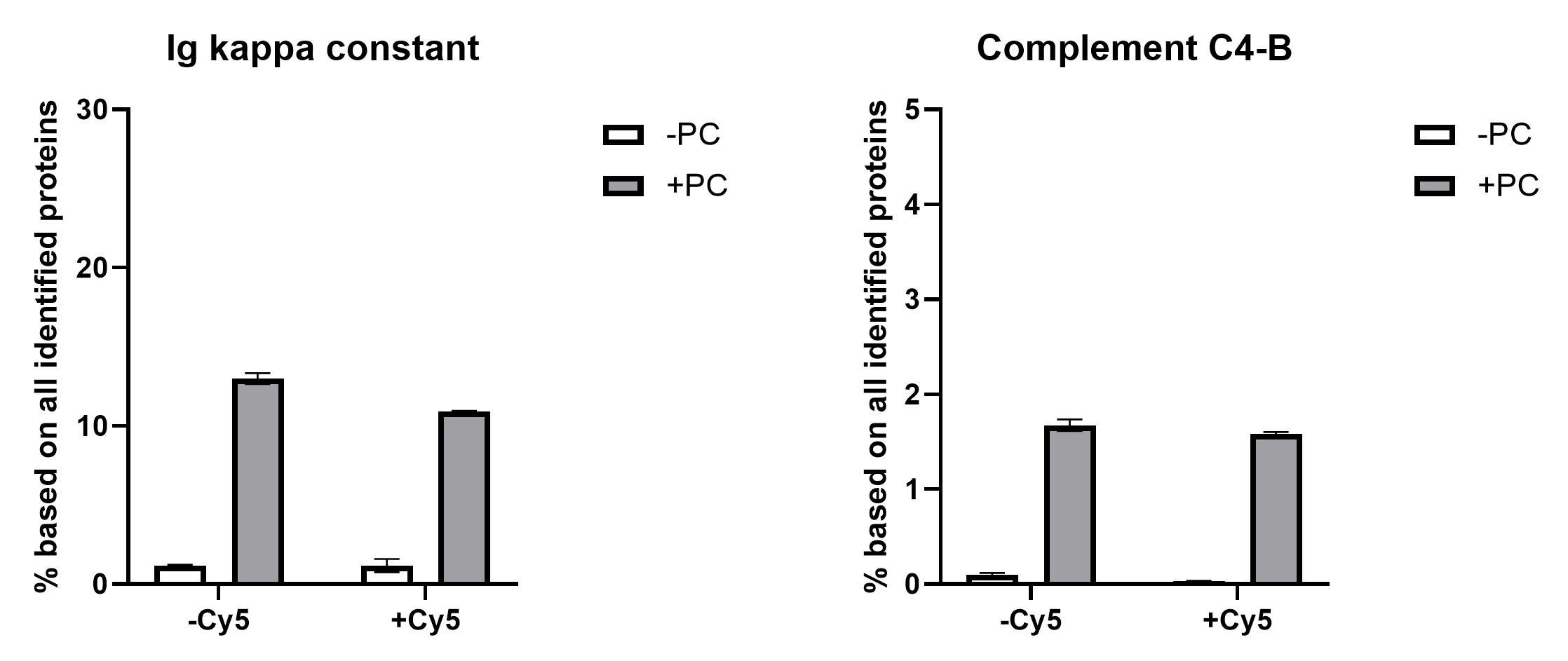


**SI figure 10:** Proteomic analysis revealed enrichment of Ig kappa constant, and complement C4-B in protein corona on EVs with and without Cy5-labeling. Mean and standard deviation of three technical replicates are shown.

**SI table 1:** Proteomic analysis of EV marker protein in nascent EV preparation.

| **UniProt Accession** | **Protein** |
| --- | --- |
| P62330 | ADP-ribosylation factor 6 |
| P04083 | Annexin A1 |
| P50995 | Annexin A11 |
| P07355 | Annexin A2 |
| P08758 | Annexin A5 |
| P08133 | Annexin A6 |
| P56539 | Caveolin-3 |
| P60033 | CD81 antigen |
| P21926 | CD9 antigen |
| P08238;Q58FF8 | Heat shock protein HSP 90-beta |
| P26006 | Integrin alpha-3 |
| P05556 | Integrin beta-1 |
| Q8WUM4 | Programmed cell death 6-interacting protein |

**SI table 2:** Proteomic analysis of plasma process control. Human plasma was incubated without EVs and subsequently centrifuged at 100 000 x g according to the protein corona adsorption procedure.

| **UniProt Accession** | **Protein** |
| --- | --- |
| P02647 | Apolipoprotein A-I |
| P10909 | Clusterin |
| P01876 | Immunoglobulin heavy constant alpha 1 |
| P01857 | Immunoglobulin heavy constant gamma 1 |
| P01871 | Immunoglobulin heavy constant mu |
| P01834 | Immunoglobulin kappa constant |
| B9A064 | Immunoglobulin lambda-like polypeptide 5 |
| P02768 | Serum albumin |
